# Stenotrophomonas maltophilia periprosthetic joint infection after reverse total shoulder arthroplasty^☆^

**DOI:** 10.1016/j.idcr.2020.e00796

**Published:** 2020-05-14

**Authors:** Michael E. Hantes, Fotios Papageorgiou, George A Komnos

**Affiliations:** The Department of Orthopaedic Surgery Faculty of Medicine, School of Health Sciences, University of Hospital of Larissa, Larissa, Greece

## Abstract

*Stenotrophomonas maltophilia* is a well-known opportunistic Gram-negative bacterium causing mainly hospital-acquired infections, **which rarely affects the musculoskeletal system**. We report the first case, to our knowledge, of a periprosthetic infection caused by this pathogen in an artificial joint. *Stenotrophomonas maltophilia* has the ability to form biofilm, and subsequently should not be excluded in the investigation of prosthetic joint infections. Management in the establishment of such an infection demands aggressive operative treatment in conjunction with the proper **antibacterial** administration.

Introduction

Reverse total shoulder arthroplasty (RTSA) has gained popularity during the past years. Indications include a wide spectrum of diseases such as cuff tear arthropathy, inflammatory osteoarthritis and failed hemiarthroplasty or fixation of proximal humeral fractures [1]. Periprosthetic joint infection(PJI) following shoulder arthroplasty has a varying incidence from 0.7 to 7% [2,3] with a **substantial** social and economic impact [3]. The predominant bacteria responsible for shoulder PJI are *Cutibacterium acnes* (**former** *Propionibacterium acnes*), ***S. aureus*, and *S. epidermidis*** [2,4,5]. Reports of musculoskeletal infection with *Stenotrophomonas maltophilia* are extremely scarce. A neutropenic patient with soleus myositis without a history of trauma has been reported in 2002 [6]. A musculoskeletal infection **affecting hand and fingers** in a non-immunosuppressed patient is the most prominent correlation between this bacterium and musculoskeletal infections [7]. Spinal procedures, such as vertebroplasty and lumbar microdiscectomy have been also demonstrated to be rarely complicated with *Stenotrophomonas maltophilia* infection [8,9].

## Case report

**Fig. 1:**
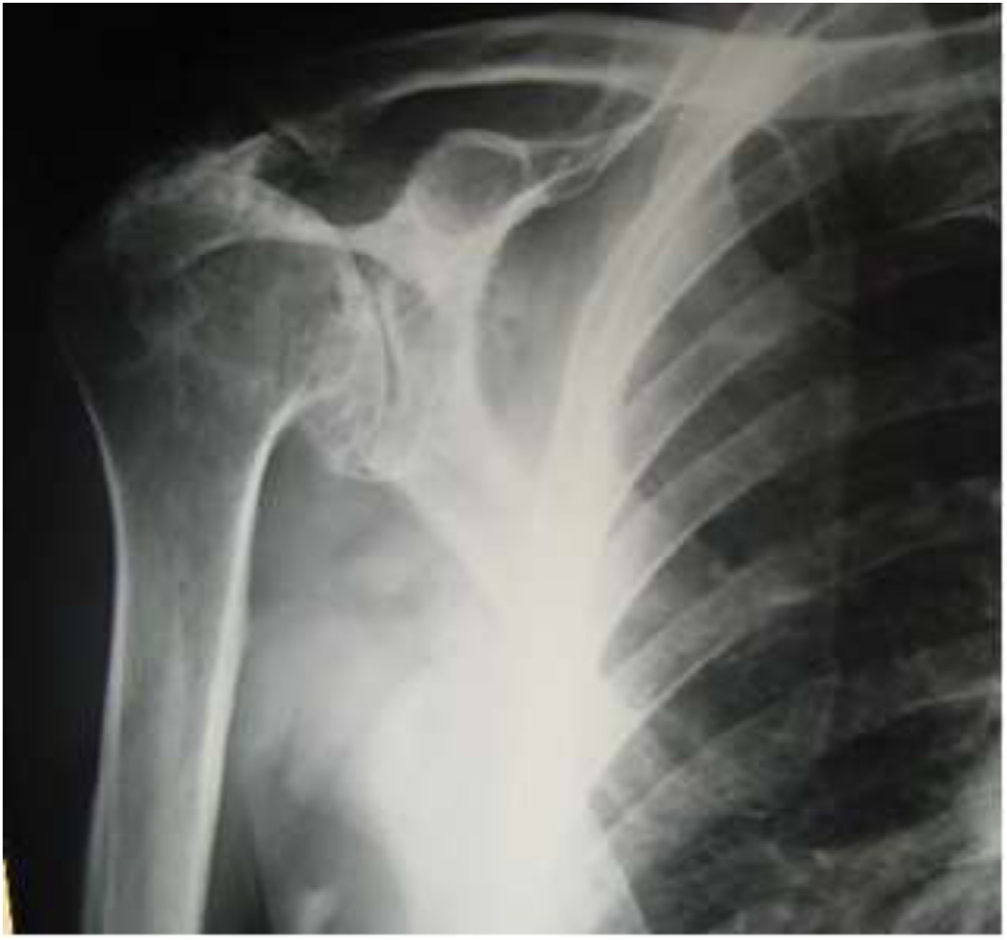
Preoperative x-ray showing the severe arthropathy.

**Fig. 2:**
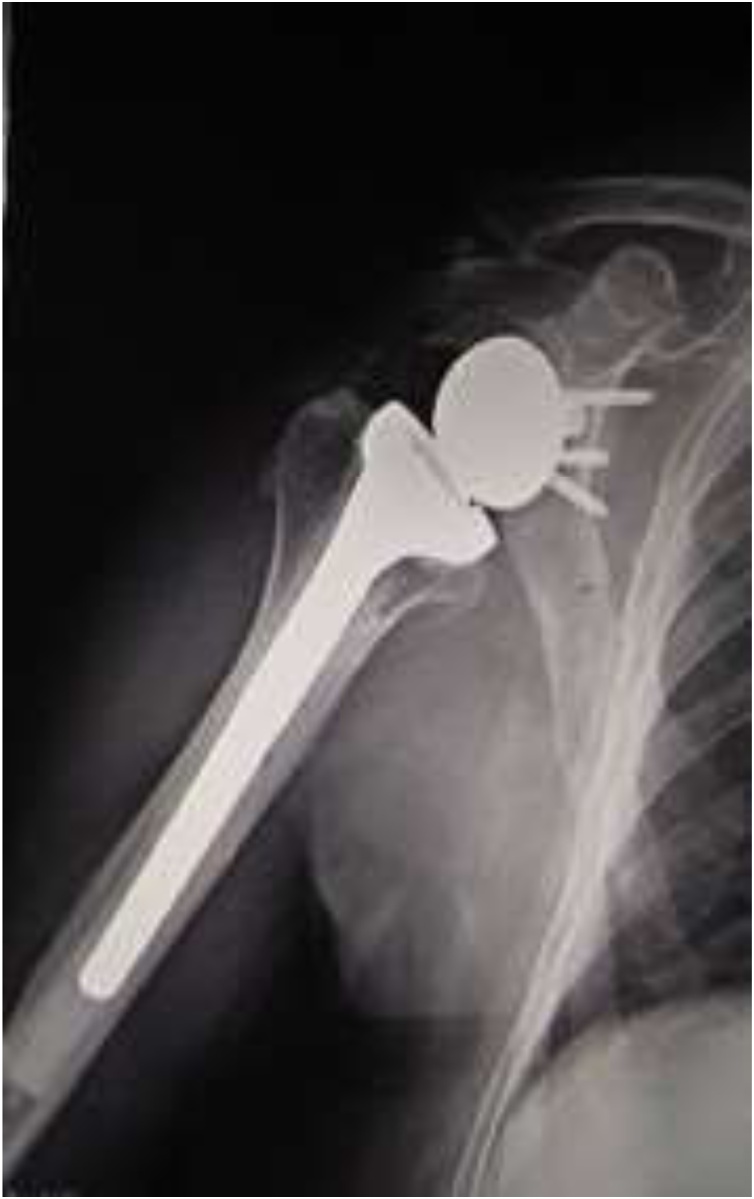
Postoperative radiographs of the RTSA.

We report a case of a **73-year-old** man who was admitted with signs of infection (edema, increased temperature, erythema) over the anterior aspect of his right shoulder. The patient had undergone a **reverse total shoulder arthroplasty** 1 year before, due to rotator cuff arthropathy (Fig. 1). Until the onset of the symptoms, the artificial joint was well functioning, with a satisfactory range of motion, no pain or other signs of infection, with radiographs demonstrating well-positioned implant with heterotopic ossification and no major osteolysis (Fig. 2). His past medical history included atrial fibrillation, arterial hypertension, and dyslipidemia under medication.

The clinical evaluation mainly, combined with mild elevation of infection markers (WBC=**12.700/ul CRP = 9.1 mg/L**, ESR = 55 mm/hr) was suggestive for deep RTSA infection. These findings along with an inconclusive joint aspiration led to an open, extended irrigation and debridement procedure. Deep tissue samples were sent for **culture**, and administration of wide spectrum **antibacterial (2nd generation Cephalosporin and aminoglycoside)** was initiated. *Stenotrophomonas maltophilia* was incubated in all 4 samples from the initial cultures and according to the antibiogram, levofloxacin and trimethoprim-sulfamethoxazole were administrated. Due to the known nature of *Stenotrophomonas maltophilia,* the patient was submitted into a full immunology status investigation (C3, C4, ANA), to find possible comorbidities, but **these were unrevealing**. Evaluating other established risk factors for the specific **bacterium**; the patient reported no corticosteroid use, no previous major infection, dental intervention or travel abroad after the RTSA. During his in-hospital stay, his condition was further complicated with acute renal failure and lower limb deep venous thrombosis (DVT). A whole body examination to exclude malignancy (chest-abdomen CT scan) was performed and proved negative. Because of these complications, cessation of **antibacterials** was decided and after signs of clinical improvement and decreasing inflammatory markers, the patient was discharged 14 days later.

**Fig. 3:**
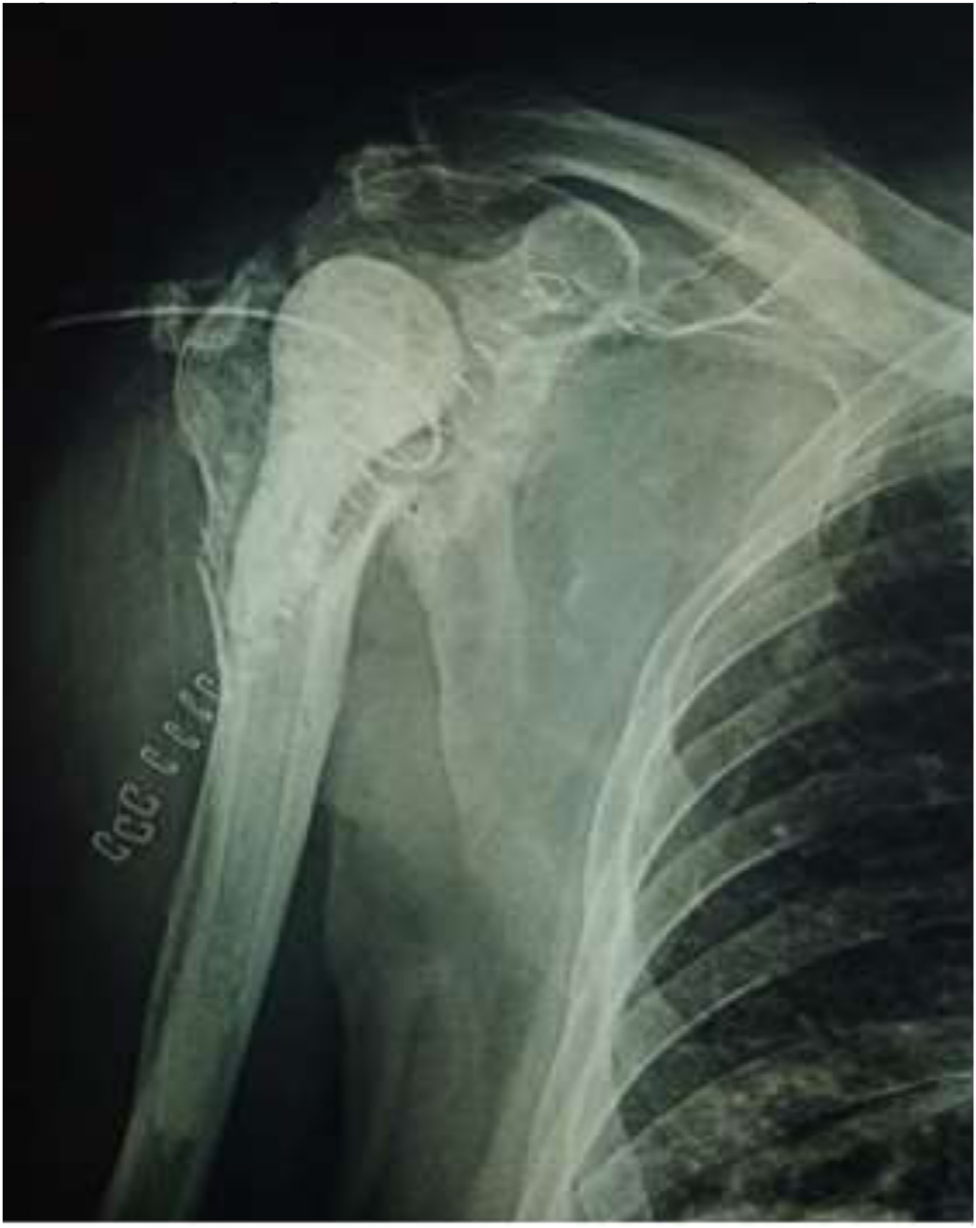
Radiograph of the shoulder in the final follow-up (30 months).

During his follow-up evaluation the patient developed a sinus tract in his joint Subsequently, two months after previous discharge, the patient was readmitted for removal of the implants with placement of **antibacterial**-**containing** cement spacer and administration of levofloxacin and trimethoprim-sulfamethoxazole i.v for 21 days (Fig. 3). **Subsequently oral** clindamycin and trimethoprim-sulfamethoxazole were administrated for another 3 weeks. Fortunately, kidney function was not compromised during this period and no further complications occurred.

Six months postoperatively, the patient reached a satisfactory functional level without major complaints and he refused the proposed second stage implantation. At the final follow up of 30 months, the patient had limited range of motion with no pain, and no reported or evident signs of infection.

## Discussion

*Stenotrophomonas maltophilia* is a well-known opportunistic Gram-negative bacterium causing mainly hospital and occasionally community-acquired infections, such as hospital acquired and ventilator associated pneumonia and bloodstream infections [10,11].

Prosthetic joint infection (PJI) in shoulder arthroplasty is underreported in the literature compared to PJI in lower limb. Most of our knowledge for the management of this type of prosthetic infection derives from the field of hip and knee arthroplasty. Regarding the microbiological profile of the pathogens causing shoulder infection in arthroplasties, it seems that *Cutibacterium acnes* (*former Propionibacterium acnes*) is the predominant one. Egglestone et al. [2] in their review identified this as the most common bacteria responsible for shoulder arthroplasty infection followed by **coagulase-negative *staphylococcus* and *Methicillin Sensitive Staphylococcus Aureus(MSSA)*.**

Reverse shoulder arthroplasty has a higher risk for infection than the conventional one [2,12]. Risk factors for shoulder PJI include male gender, younger age, diabetes mellitus (DM) and high BMI [5,12,13]. Our patient although male, he was not young, neither suffered form DM or high BMI. Regarding *S.maltophilia* specifically, hematologic malignancy is associated with increased risk for infection from this pathogen [11]. A complete investigation in our case revealed no such underlying disease though. Furthermore, one of the remarkable issues in our patient is that none of the other cited risk factors for *S. maltophilia* infection were present. No previous intensive care admission, no steroid use, and no neutropenia [14,15].

Report of ***S. maltophilia*** infections in **orthopedic** literature is extremely scant. Spine surgery has been demonstrated to be complicated with infection caused by this pathogen. In particular, isolated, scattered cases have been published [9]. In the most recent of them, a non-immunocompromised patient developed epidural pus following a lumbar microdiscectomy, which was successfully treated with 6 weeks of intravenous cefoperazone-sulbactam followed by 6 weeks of oral levofloxacin. Few cases of skin infections have also been reported in healthy patients or patients with concomitant diseases [7,16]. Nevertheless, **antibacterial** treatment with trimethoprim/ sulfamethoxazole along with surgical debridement was eventually effective in a case of myositis [6].

As far as we are aware of, this is the first case in the literature that ***S.maltophilia*** causes infection in a prosthetic joint. It would be equitable to claim that the low virulence of this pathogen precludes its ability to form biofilm. However, it is established that *S. maltophilia* has the ability to form biofilm in several biotic and abiotic surfaces [15]. Additionally, it has been demonstrated that *S. maltophilia* can form biofilms either on its own, or in synergy with other species; outstandingly, once growing in biofilms it is more resistant to phagocytes and **antibacterials** [11]. Most likely, this low virulence of the pathogen is that makes it absent, until now, from reported prosthetic infections.

Treatment options for shoulder PJI are identical to hip and knee arthroplasty; one or two-stage revision, debridement, resection arthroplasty, or arthrodesis [17]. Treatment, infection from *S maltophilia* occurs, is extremely challenging because of the high level intrinsic resistance of this pathogen. **Trimethoprim/sulfamethoxazole** alone, or in combination with other agents, is still considered the treatment of choice *S maltophilia* infection. Isolation of the infecting pathogen allows the administration of organism-specific **antibacterials**, increasing the chances of eradicating the infection. In our case, the patient was satisfied with the level of function of his shoulder; therefore no re-implantation was performed. Nevertheless, resection arthroplasty has revealed to offer good pain relief, and only slightly worse functional results than two-stage exchange procedures [17].

To our knowledge, this is the first reported case of **total joint arthroplasty** infection caused by *Stenotrophomonas maltophilia* in a non-immunocompromised patient. It should be kept in mind of **orthopedic** surgeons as possible rare infectious microorganism that requires combined approach for the operative treatment and the optimal **antibacterial** choice.

## Author contribution

Michael Hantes: study design, data collection, data interpretation, manuscript writing

Georgios Komnos: data collection, data analysis, figures, manuscript writing

Fotios Papageorgiou: data collection, data analysis, figures, manuscript writing

## Sources of funding

No funding.

## Consent

"Written informed consent was obtained from the patient for publication of this case report and

accompanying images. A copy of the written consent is available for review by the Editor-in-Chief of this journal on request”.

## Declaration of Competing Interest

No conflicts of Interest.
